# The retinoic acid response is a minor component of the cardiac phenotype in H9c2 myoblast differentiation

**DOI:** 10.1186/s12864-023-09512-0

**Published:** 2023-08-02

**Authors:** Carlos Campero-Basaldua, Jessica Herrera-Gamboa, Judith Bernal-Ramírez, Silvia Lopez-Moran, Luis-Alberto Luévano-Martínez, Hugo Alves-Figueiredo, Guillermo Guerrero, Gerardo García-Rivas, Víctor Treviño

**Affiliations:** 1 Tecnologico de Monterrey, Escuela de Medicina y Ciencias de la SaludCátedra de Bioinformática, Ave. Morones Prieto 3000, Colonia Los Doctores, Monterrey, Nuevo León 64710 Mexico; 2 Tecnologico de Monterrey, Escuela de Medicina y Ciencias de la SaludCátedra de Cardiología y Medicina Vascular, Hospital Zambrano Hellion, San Pedro Garza García, P.C. 66278, Monterrey, Nuevo León 64710 Mexico; 3 Tecnologico de Monterrey, The Institute for Obesity Research, Eugenio Garza Sada Avenue 2501, Monterrey, Nuevo Leon 64849 Mexico

**Keywords:** H9c2 cell line, Myoblast, Myotube, Transcriptomic, Epigenomic

## Abstract

The H9c2 myoblast cell line, isolated from the left ventricular tissue of rat, is currently used in vitro as a mimetic for skeletal and cardiac muscle due to its biochemical, morphological, and electrical/hormonal signaling properties. During culture, H9c2 cells acquire a myotube phenotype, where a critical component is the inclusion of retinoic acid (RA). The results from some authors on H9c2 suggested that thousands of genes respond to RA stimuli, while others report hundreds of genes responding to RA over different cell types. In this article, using a more appropriate experimental design, we first confirm the H9c2 cardiac phenotype with and without RA and report transcriptomic and physiological changes regarding calcium handling, bioenergetics, and other biological concepts. Interestingly, of the 2360 genes showing a transcriptional change, 622 genes were statistically associated with the RA response. Of these genes, only 305 were RA-specific, and the rest also showed a culture-time component. Thus, the major expression changes (from 74 to 87%) were indeed due to culture conditions over time. Unexpectedly, only a few components of the retinol pathway in KEGG responded to RA. Our results show the role of RA in the H9c2 cultures impacting the interpretation using H9c2 as an in vitro model.

**Supplementary Information:**

The online version contains supplementary material available at 10.1186/s12864-023-09512-0.

## Introduction

The H9c2 myoblast cell line, isolated from the left ventricular tissue of Rat, is currently used in vitro as a mimetic for skeletal and cardiac muscle due to its biochemical, morphological, and electrical/hormonal signaling properties [1–3]. In this stage, cells are still not fully differentiated into adult cardiomyocytes but are already predestinated, leading to the appearance of several cardiomyocyte-specific markers [2, 3]. An essential feature of this embryonic cell line is its ability to differentiate from mono-nucleated myoblasts to myotubes when the H9c2 cells were cultured in a low serum concentration media [2–4]. During the differentiation process, cells obtain mainly a skeletal muscle phenotype, as evidenced by cell type-specific differentiation markers [5]. Furthermore, different authors demonstrated that the addition of all-trans retinoic acid (RA) to a 1% serum media induces a predominant presence of cells presenting an adult cardiac muscle phenotype, characterized by the overexpression of the alpha-1 subunit of L-type calcium channels [5], cardiac proteins as troponin I, troponin T, calsequestrin, and different mitochondrial proteins [1]. It has been shown that H9c2 cells do not present contractile activity, even when differentiated [1, 2, 4]. However, rat H9c2 cells were isolated from the left ventricle and, compared with isolated neonatal cardiomyocytes, they responded similarly to several stimuli, including the development of hypertrophic responses [6, 7]. Nevertheless, most studies in the last years have been performed using undifferentiated H9c2 myoblasts cells, raising questions on the importance and relevance of the different results obtained compared with cardiomyocytes [1]. Moreover, murine studies have shown that around 350 DNA loci respond to RA in a particular cell and more than 700 over different cell types [8]. These contrast with the results that between 3400 and 5100 genes were found differentially expressed in H9c2 during the RA stimuli [1, 9].

It has been known that retinoic acid (RA) is derived from liposoluble vitamin A (retinol), and its action was elucidated as an acidic metabolite that acts as a ligand for transcription factors of the nuclear retinoic acid receptor (RAR), switching them from potential repressors to transcriptional activators [10]. RA also improves the generation of oocyte-like cells (OLCs) from mouse skin-derived somatic stem cells and its ability as a co-activator of different nuclear transcriptional activators [11]. Moreover, several cardiac markers, such as troponins I and T, have been mainly linked to the RA response [1]. Although some efforts have been made to characterize the H9c2 molecular state [1, 9], their estimation of response genes is not fully explained by RA [8]. Thus, no specific experimental design and molecular characterization have shown the detailed components involved in the H9c2 cardiac phenotype, particularly in response to RA.

In the present work, we studied the H9c2 after 1% serum with and without RA for 14 days. We first confirm the phenotype described in previous studies and then demonstrate that the overall transcriptional response during H9c2 differentiation of myoblast to myotube cells as a cardiac phenotype is mainly due to culture time rather than to RA. Our results suggest that cells are indeed predestinated to cardiac phenotype and provide information on the precise response to RA stimuli. These data will help disentangle the contribution of RA and time, impacting the interpretation of past and future results.

## Material and methods

### Cell culture

The H9c2 cell line obtained from ATCC (Manassas, VA, USA) was cultured in Dulbecco’s modified Eagle’s medium (DMEM) (SIGMA), supplemented with 3.7 g/L sodium bicarbonate (SIGMA S5761), 10% fetal bovine serum (FBS), 100 U/ml penicillin, and 100 μg/ml streptomycin in 75 cm^2^ tissue culture flasks at 37 °C in a humidified atmosphere of 5% CO2. Cells were fed every 2–3 days and subcultured when reaching 70–80% confluence to prevent the loss of the differentiation potential. Cell differentiation was initiated by H9c2 cells at a density of 35,000 cells/ml in 25 cm^2^ tissue culture flasks for 24 h for cell attachment. Then, decreasing FBS from 10 to 1% in the media, followed by Retinoic Acid (RA) supplementation [1, 5]. The RA (1 μM) addition was performed daily in dark conditions for 14 days. All-trans-RA was prepared in DMSO and stored at -20 °C in dark conditions to avoid degradation [1]. For Ca2+ signaling experiments, cells were plated in 6-well plates with coverslips at a density of 15,000 cells/ 400 µL. Cells were incubated at 37 °C for 30 min, afterward added 600 µL of DMEM supplemented with 10% FBS. After 24 h, FBS was decreased to 1% and treated with 1 μM daily for 7 or 14 days, as previously stated.

### Calcium signaling in H9c2 cells

H9c2 cells were grown on a coverslip glass, and differentiation conditions were initiated 24 h after being seeded. Prior to loading with fluorescent dyes, the cells were preincubated for 3 min in Tyrode solution (120 mM NaCl; 5 mM KCl; 0.4 mM NaH2PO4; 0.5 mM MgCl2; 25 mM HEPES; 1 mM CaCl2; 0.5 mM glucose, pH 7.4) and loaded with 10 µM Fluo-4 for 45 min at 37 °C in dark conditions. Afterward, coverslips were rinsed with fluorophore-free Tyrode and mounted in a perfusion chamber. Ca2+ transients were evoked by perfusion of caffeine (20 mM, 58-08-2) followed by perfusion of vasopressin (100 nM, V9879) [12]. Fluorescent images were acquired using a Leica TCS SP5 II epifluorescence confocal microscope (Leica Microsystems, Wetzlar Germany), using a 40× oil immersion objective, and analyzed by ImageJ software (http://imagej.nih.gov/ij/, NIH, Bethesda, MD, USA). Fluorescence data is shown as ΔF/F0, where F0 is the average fluorescence intensity before caffeine perfusion.

### Oxygen consumption and mitochondrial mass

At the indicated times, cells were detached, washed in PBS, and manually counted in a hemocytometer. Cells were suspended in respiration buffer (110 mM sucrose, 10 mM KH2PO4, 3 mM MgCl2, 0.5 mM EGTA, 60 mM Potassium gluconate, 20 mM HEPES pH 7.4). Cells were diluted inside at a final concentration of 5 × 104 cells/mL inside the chambers of an Oroboros High-Resolution oxygraph (Innsbruck, Austria). Once the basal lecture stabilized, digitonin (5 µg/mL) was added to permeabilize the cells. Succinate (20 mM) / rotenone (0.5 µM) were added as respiratory substrates. After the basal respiration stabilizes, 3 µM antimycin A was added to obtain non-mitochondrial respiration. Cytochrome c oxidase (Cox) activity was measured polarographically in permeabilized cells inhibited with antimycin (3 µM). Ascorbate (2 mM) and TMPD (100 µM) were used as Cox substrates. At the end of the experiment, 50 µM potassium cyanide was included to obtain the non-specific reduction of TMPD. Citrate synthase activity was measured in total homogenates, as reported [13]. The total protein in homogenates was determined by the Lowry method.

### Total RNA extraction

The collection of total RNA extracts was performed following the TRIzol™ reagent protocol instructions (Invitrogen). Add 0.3–0.4 mL of TRIzol™ reagent per 1 × 10^5^–10^7^ cells directly to the culture dish to lyse the cells. The suspension was homogenized, pipetting the lysate up and down several times and incubating for 5 min at room temperature to permit the complete dissociation of the nucleoproteins complex. After this time, 0.2 mL of chloroform was added per 1 mL of TRIzol™ reagent used for lysis and incubated for 3 min on ice. Samples were centrifuged for 15 min at 12,000 × *g* at 4 °C, and the aqueous phase that contained the RNA was transferred to a new tube. For the RNA precipitation, we added 0.5 mL of isopropanol to the aqueous phase per 1 mL of TRIzol™ reagent used for lysis, and the sample was incubated for 10 min on ice. The samples were then centrifuged for 10 min at 12,000 × *g* at 4 °C. The supernatant was discarded, and the pellet was resuspended in 1 mL of 75% ethanol per 1 mL of TRIzol™ reagent used for lysis. The sample was vortexed for 5 s and immediately centrifuged for 5 min at 7500 × *g* at 4 °C; the supernatant was discarded, and the RNA pellet was dried for 10 min on ice. Pellet was resuspended in 50 µL of RNase-free water. Total RNA was quantified using a UV analysis Synergy™ HT Multi-Detection Microplate Reader (Biotek Instruments). To examine RNA integrity and DNA contamination, 0.5 μg of RNA was electrophoretically separated in an agarose gel. Samples were then saved at -80 °C.

### RT-q-PCR assay

RNA was obtained from cells grown in 25 cm^2^ tissue culture flasks at 0, 7, and 14 days under differentiation conditions (1% FBS + RA) and cells grown at the same times/days in non-differentiation conditions (1% FBS) (as control). Cells were treated directly with TRIzol™ Reagent (INVITROGEN); cDNA synthesis was performed using the SensiFast cDNA synthesis kit (BIOLINE). For gene expression profile, we designed oligonucleotides (Fw. 5ʹ AGC TCA AAT TCA CTG CCA AGC 3ʹ and Rv. 5ʹ ACA ATG TCT GTG GCC ACG TT 3ʹ) to amplify Ca^+2^ Channel L-type α1c (Cacna1c); (Fw. 5ʹ CCC ACC TCC TTG ACA TCG 3ʹ and Rv. 5ʹ CAG CCA ACA AGC CAA CAG 3ʹ) to amplify Ryanodine receptor-2 (RyR2); (Fw. 5ʹ GCA GTG CGT GTT TGT TGC TA 3ʹ and Rv. 5ʹ CAA TTT GGG GTT CTC ATG CTT G 3ʹ) to amplify Ca^+2^ Channel L-type α1s (Cacna1s); (Fw. 5ʹ TTCATC AGC CTT TCC CCA CC 3ʹ and Rv. 5ʹ CTC CAG ACA CCG AGT CCC TA 3ʹ) to amplify the transcriptional factor E2f1 and (Fw. 5ʹ ACC CAG AAG ACT GTG GAT GG 3ʹ and Rv. 5ʹ ACA CAT TGG GGG TAG GAA CA 3ʹ) to amplify (GAPDH) as control. qPCR reactions were performed using the SensiFast SyBR Lo-Rox Kit (BIOLINE), and the samples were running in a QuantStudio 3 real-time PCR system (THERMO FISHER) by following the next conditions: 95 °C-30 s, 61 °C-30 s and 72 °C-1 min × 40 Cycles.

### Western blot assay

H9c2 cells were trypsinized and centrifuged for 5 min at 700 × g, and the pellet was washed with cold PBS. Cells were lysed by resuspension in RIPA buffer containing sodium orthovanadate, a phosphatase inhibitor, supplemented with 2 mM DTT, 100 μM PMSF, and a protease inhibitor cocktail. Extracts were sonicated at 60% amplitude 1 pulse of 15 s followed by 1 min on ice × 3 times and stored at -80 °C until used. Protein contents were determined by the Lowry and Bradford method [14, 15] using bovine serum albumin as standard. After denaturation for 5 min at 95 °C in Laemmli buffer 1× supplemented with β-mercaptoethanol, 25 µg of protein of each sample was separated by electrophoresis in 10% or 12% SDS-polyacrylamide gels (SDS-PAGE) and transferred to a polyvinylidene difluoride (PVDF) membrane. After blocking with 5% milk or BSA in PBST (50 mM Tris-HCl, pH 8; 154 mM NaCl and 0.1% tween 20) for 2 h at room temperature or overnight at 4 °C, membranes were incubated overnight at 4 °C with the specific antibodies: mouse monoclonal anti-Calcium channel L-Type (Cacna1a) (1:1,000), mouse monoclonal anti-Calcium channel skeletal-Type (Cacna1s) (1:1,000), the membranes were washed with PBST 1 × 10 min for 3 times and incubated at room temperature for 2 h with goat anti-mouse (1:5,000) secondary antibodies conjugated from Cell Signaling (Danvers, MA, USA). Membranes were incubated with SuperSignal™ West DuraExtended Duration Substrate detection reagent (Thermo Scientific) and imaged using the ChemiDoc XRS+ System imaging system (Bio-Rad). The density of different bands was calculated with Image J Software 1.50a (NIH, USA). Ponceau staining was used to confirm equal protein loading and to normalize the data, as the differentiation process can affect the number of housekeeping proteins, such as beta-actin. Original raw gel images are available in the supplementary information.

### Cell area and multinucleated cells analysis

The cellular area and counting of polynucleated cells following cell-differentiation protocol were performed in live cells by fluorescence using calcein-AM to stain against cell cytoplasm and DRAQ to stain nuclei. Briefly, live cells seeded in coverslips were washed with Tyrode 1x (120 mM NaCl; 5 mM KCl; 0.4 mM NaH2PO4; 0.5 mM MgCl2; 25 mM HEPES; 0.5 mM glucose, pH 7.4), and then incubated at 37 °C in a 5% CO2 and 95% air-humidified atmosphere for 30 min, with a 1 µM calcein (Invitrogen, MA, USA). Afterward, coverslips were mounted in a perifusion chamber, and immediately prior to acquiring fluorescent images, cells were incubated for 5 min with nuclei-staining probe Draq5™ (10 μM, Thermofisher Scientific, MA, USA). Fluorescent Images were acquired using a Leica TCS SP5 II epifluorescence confocal microscope (Leica Microsystems, Wetzlar Germany), using a 40× oil immersion objective. The fluorescence calcein was assessed using an Ar laser at 488 nm, and emission was acquired at 517 nm with a bandwidth of 20 nm; DRAQ5 was assessed using a He-Ne laser at 633 nm (excitation), and emission was acquired at 733 nm with a bandwidth of 66 nm. The area of positive Calcein fluorescence (surrogate of cell area), as well as the number of nuclei, was determined for each cell. Image analysis was performed using ImageJ software (1.50a; http://imagej.nih.gov/ij/, NIH, Bethesda, MD, USA).

### RNA-seq

35 × 10^3^ to 1 × 10^6^ of H9c2 cells were plated in 25 cm^2^ tissue culture flasks at 0, 7, and 14 days and induced to differentiation conditions as described above. Cells were treated directly with TRIzol™ Reagent (Invitrogen), and RNA was quantified with a Qubit RNA High Sensitivity assay kit (Thermo Fisher Scientific). Sequencing library preparation was carried out using cDNA synthesis for next-generation sequencing (NGS) performed by TruSeq Stranded mRNA Sample Preparation Kit (Illumina, San Diego, CA, USA). Paired-end sequencing (2 × 75 bp reads) was done by three biological replicates for each of the three points. Library sequencing was performed using a MiSeq Reagent Kit v3 in a MiSeq Sequencer (Illumina) following the manufacturer’s instructions.

### Quantification and statistical analysis

To process RNA-Seq data, we used the Galaxy platform (http://usegalaxy.org). Briefly, fastqz files were uploaded, QC cleaned, trimmed, mapped to rat genome using *HiSat2*, and gene counted by *FeatureCounts*. The counts are available in GEO at NCBI by the id GSE221596. Of the 32,883 annotated transcripts, 15,953 did not show any transcripts. Thus, only transcripts summing up more than 20 read counts among the 15 samples were considered. Finally, 11,574 Ensembl annotated rat transcripts were used. A limma [16] model was used to identify culture time and RA statistically associated factors (expression ~ t7 + t14 + RA + b). The *p*-values for each factor were corrected by the false discovery rate [17]. The minimum q-value of the T7, T14, or RA was used to determine differential expression. Only q-values < 0.001 were considered resulting in 2360 genes. For the calcium signaling, the data process was performed using Microsoft Excel and GraphPad Prism 8.0. Data were considered statistically significant when *p* < 0.05. GraphPad Prism 8.0 software was used to generate graphs to present results. The area under the curve (AUC) was performed to analyze Ca2+ transients. One-way and Two-way ANOVA were used to determine changes between groups. Data were expressed as means ± SEM.

## Results

### Characterization and validation of the cardiac phenotype

**Fig. 1:**
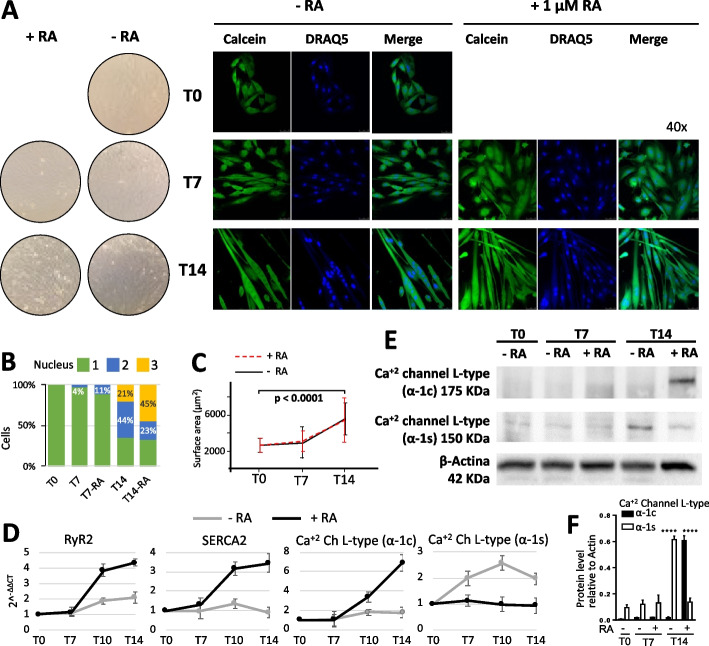
Morphological and molecular analysis. **A** H9c2 cellular morphology by bright-field microscopy (left) and H9c2 cells loaded with calcein (cytoplasm, green) and DRAQ5 (nuclei, blue) (right) with and without the presence of retinoic acid. **B** Percentage of cells with one, two, or 3 or more nuclei. **C** Pooled data of cell surface area. **D** Gene expression semi-quantification of selected proteins in calcium signaling by qPCR analysis. **E** Protein expression by Western blot of cardiac (a) and skeletal (s) subunits of L-type Ca2+ channel, b-actin used as the loading control. **F** Semi-quantification plot of the expression of cardiac (α-1c) and skeletal (α-1s) subunits of L-type Ca2+ channel assessed by Western Blotting. In all panels, T0 refers to 24 h after seeding, T7 to 7 days after seeding, and T14 to 14 days after seeding. *N* = 3 for **B**, **C**, **D**, **E**, and **F**; 450 cells analyzed for each experiment in **B** and **C**. Data shown as avg ± sem. Statistics: two-way ANOVA, ****, *p* < 0.0001 +RA: with retinoic acid; -RA: without retinoic acid

Branco and others have used a 5 to 6 days H9c2 differentiation protocol using 1% serum and 1uM RA [1, 9]. Here we controlled for the variables time and RA treatment by analyzing cells in cultures with and without RA and extending both cultures to 14 days. First, we confirmed the elongated shape, partially multinucleated, and viability in 7 and 14 days (Fig. 1A). We noted an increase in bi-nucleated cells at 7 days of exposure to RA from 4 to 11% (Fig. 1B). Remarkably, at 14 days, cells with RA presented a 2-fold increase in the number of multinucleated cells when compared to control, but no differences in the number of mononucleated cells between both groups (Fig. 1B). Along with this, it was also observed that cell surface increased along culture time regardless of the use of RA (Fig. 1C). To characterize the cardiogenic phenotype, instead of Troponin I and T used by Branco et al., which will be detailed in further sections, we measured by qPCR the genes Serca2, RyR2, the cardiac-specific calcium channel type-L α1C, and the skeletal specific calcium channel type L α1S [18]. We observed a sustained relative increase in RyR2, Serca2, and cardiac Ca+ channel α1C under RA with time, while the skeletal Ca+ channel α1S increased in the absence of RA (Fig. 1D-F). In summary, we confirmed the differentiation of cells towards a cardiac myotube through analysis of the overall morphology and expression markers of H9c2 cells under RA treatment and highlighted differences relative to cells without the RA treatment at the same culture times.

### RA treatment influences functional cardiac responses

**Fig. 2:**
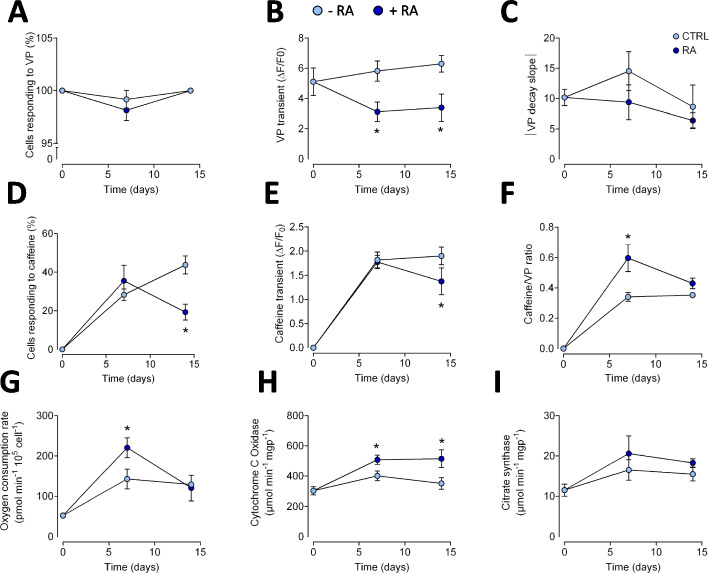
Intracellular Ca^2+^ dynamics and metabolism in H9c2 under RA and control H9c2 cells. **A** Percentage of responsive cells to vasopressin. Pooled data of vasopressin-induced transient amplitude (**B**) and the absolute transient decay slope (**C**). **D** Percentage of responsive cells to caffeine. **E** Pooled data of caffeine-induced transient amplitude. **F** Pooled data of the ratio between caffeine- and vasopressin-induced transient amplitude. **G** Mitochondrial oxygen consumption rate in permeabilized cells stimulated with succinate and rotenone. **H** Cytochrome *c* oxidase activity measured polarographically in permeabilized cells. **I** Mitochondrial mass was determined by the activity of citrate synthase in cell homogenates. +RA: with retinoic acid; -RA: without retinoic acid; In all panels, T0 refers to 24 h after seeding, T7 to 7 days after seeding, and T14 to 14 days after seeding. *N* = 4–7 for **A**-**F**, 30 cells analyzed in each experiment. *N* = 3 for **G**-**I**, 350 000 cells analyzed in each experiment. Statistics: two-way ANOVA analysis. * *p* < 0.05 vs. its respective time control

We performed a functional analysis of calcium handling and cellular respiration in both control and RA-treated groups over time. To assess the calcium dynamics, Ca^2+^ release was induced using caffeine or vasopressin to stimulate RyR or IP3 receptor (IP3R) opening, respectively. Despite treatments, all groups maintained their responsiveness to vasopressin (T0: 100%, T7: 99.2%, T14: 100%, T7 + RA: 98.1%, T14 + RA: 100%) (Fig. 2A). While vasopressin-induced transient amplitude remained unchanged in the control group (T0: 5.10 ΔF/F0, T7: 5.82 ΔF/F0, T14: 6.29 ΔF/F0), treatment with RA showed a mild decrease (T0: 5.10 ΔF/F0, T7 + RA: 3.12 ΔF/F0, T14 + RA: 3.39 ΔF/F0), with important differences among treatments, indicating a loss of IP3R activity (Fig. 2B). Regarding caffeine responsiveness, 24 h after seeding, no cell responded to caffeine perfusion; however, over time, the control group increased the percentage of cells’ responsiveness to caffeine (T7: 28%, T14: 44%, Fig. 2D), indicating an increase in the presence and function of RyR. The RA-treated group showed a peak in caffeine responsiveness on day 7 (36%) that, importantly, decreased on day 14 (19%, Fig. 2D). Caffeine-transient amplitude was similar in all groups (T7: 1.82 ΔF/F0, T7 + RA: 1.77 ΔF/F0, T14: 1.9 ΔF/F0) with a slight decrease at day 14 under RA (T14 + RA: 1.37 ΔF/F0) (Fig. 2E). The caffeine-vasopressin amplitude ratio in the control group showed an increase on day 7, that is maintained on day 14 (T7: 0.34, T14: 0.35); while RA treatment showed a clear increase on day 7, suggesting an increase in Ca^2+^ handling by RyR (T0: 0, T7 + RA: 0.60); however, on day 14 this ratio decays showing no difference between groups (T14 + RA: 0.43, Fig. 2F). This data suggest that time differentiates cells in a more consistent way, while RA treatment causes rapid cell degradation, being culture time the main component for a cardiac-like phenotype differentiation in H9c2 myoblast. On the other hand, cellular respiration increases independently of the treatment with RA. However, a higher but transient increase in oxygen consumption was observed after 7 days in RA-treated cells. This same trend was observed with the activity of Cox, the terminal enzyme of the respiratory chain. Interestingly, this increase was not parallel with the activity of citrate synthase, an established marker of mitochondrial mass. This result rules out the effect of RA on mitochondrial biogenesis. Moreover, this result indicates that differentiation time, and not RA per se, is the causal agent of this transient increase in respiratory activity.

### Transcriptional response of H9c2 cells over time and RA during differentiation

**Fig. 3:**
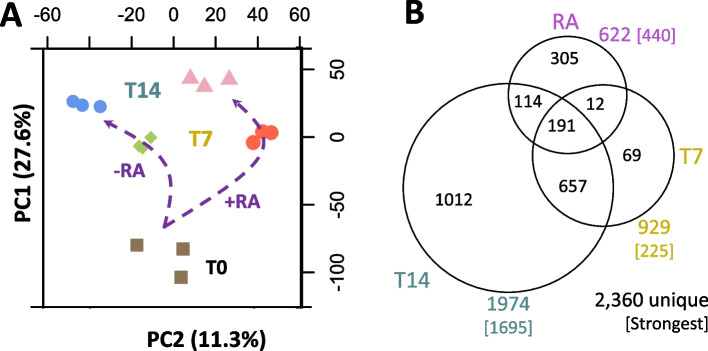
Overall transcriptional changes. **A** Representation of the first two principal components from the transcriptional profiles, which correspond to time (PC1, increasing vertically from T0 to T14) and RA treatment (PC2, without RA at the left and with RA at the right). **B** The number of genes significantly associated with specific factors. For example, for RA, there are 305 genes specifically associated to RA only, 12 shared with those changing at 7 days, 114 shared with those genes changing at 14 days, and 191 shared with both 7 and 14 days. Also, there were 622 genes in total associated to RA but only 440 where the strongest factor is indeed RA. PC1: principal component 1; PC2: principal component 2; RA: retinoic acid; +RA: with retinoic acid; -RA: without retinoic acid; T0: 24 h after seeding; T7: 7 days after seeding; T14: 14 days after seeding

**Fig. 4:**
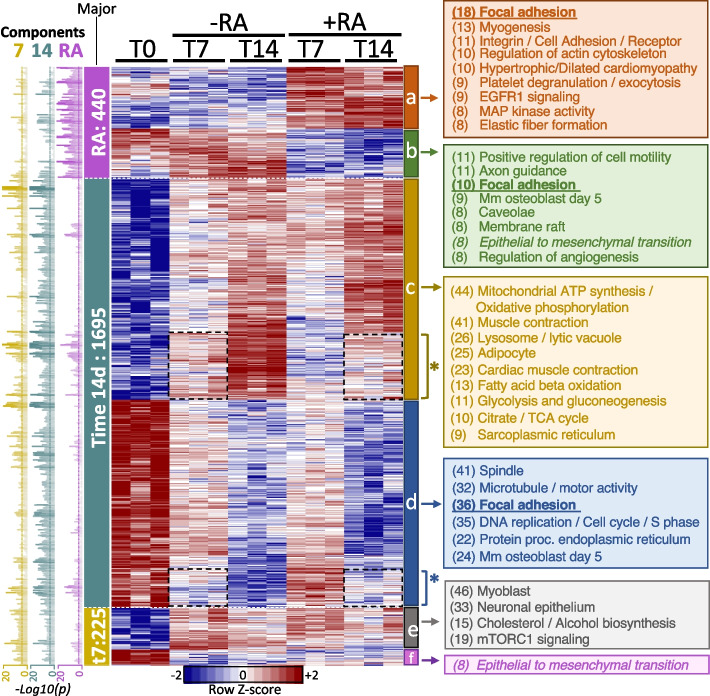
Genes statistically associated with time and RA, including a functional analysis. Heatmap shows the gene expression level in z-score. The *p*-value associated with specific time and RA factors is shown on the left (7 and 14 refers to days). Genes were first associated with the primary factor by the most significant *p*-value and then clustered by gene expression profiling. The clusters a-f formed were analyzed by functional enrichment as indicated by block colors showing the results at the right where the numbers within parenthesis define the number of genes associated to the functional term. Asterisk (*) marks “delayed” genes referred to in the text where gene expression levels are similar in 7 days without RA and 14 days with RA. +RA: with retinoic acid; -RA: without retinoic acid; T0: 24 h after seeding; 7 and T7 or t7: 7 days after seeding; time 14 and T14: 14 days after seeding

To generate a panorama of the main components involved in the differentiation process and to provide details of the changes, we performed a transcriptomic analysis using RNA-Seq in three biological replicates of H9c2 cells with or without RA for 14 days. After normalization, a principal component analysis showed that, overall, the top two main components correspond to time (PC1) and RA treatment (PC2), explaining 27.6% and 11.3% of the variance, respectively (Fig. 3A). This result suggests that culture time is the major component of the differentiation process. We then used a linear multifactorial model (limma, see Materials and methods) to consider time and RA treatment and used coefficient-specific *p*-values adjusted for multiple tests as selection criteria (q < 0.001). We found 2,360 unique genes changing their expression relative to the baseline at time 0. Most of the genes changed on day 14 (1974), while 929 genes were detected on day 7, and 622 were affected by RA irrespective of time (Fig. 3B). Because many genes show significance to both factors, we used the lowest *p*-value to determine the most influential factor. Correspondingly, 1695 genes on day 14 (71.8%) and 225 genes on day 7 (9.5%) matched time as their more critical contributor, whereas only 440 genes (18.6%) were more assertively associated with RA. We used the main factor and the sign of the change to group genes in clusters (from cluster *a* to cluster *f* in Fig. 4) and analyzed their gene expression patterns and functional properties (Fig. 4). We first noted that genes whose strongest factor was RA (clusters* a* and *b*) and 7 days (clusters *e* and *f*) showed abrupt and sustained gene expression changes, while genes designated on day 14 showed gradual changes (clusters *c* and *d*). This result indicates that the RA response appears early and is persistent. A small subset of genes more associated with day 14 also show a minor contribution from the RA treatment (marked with * in Fig. 4), where the gene expression level of day 14 in RA resembles the expression level at day 7 without RA. Correspondingly, day 7 in RA appears as a middle point between day 0 and day 7 without RA. These results suggest a “delay effect” in these genes.

### Functional analysis of the transcriptional response

**Fig. 5:**
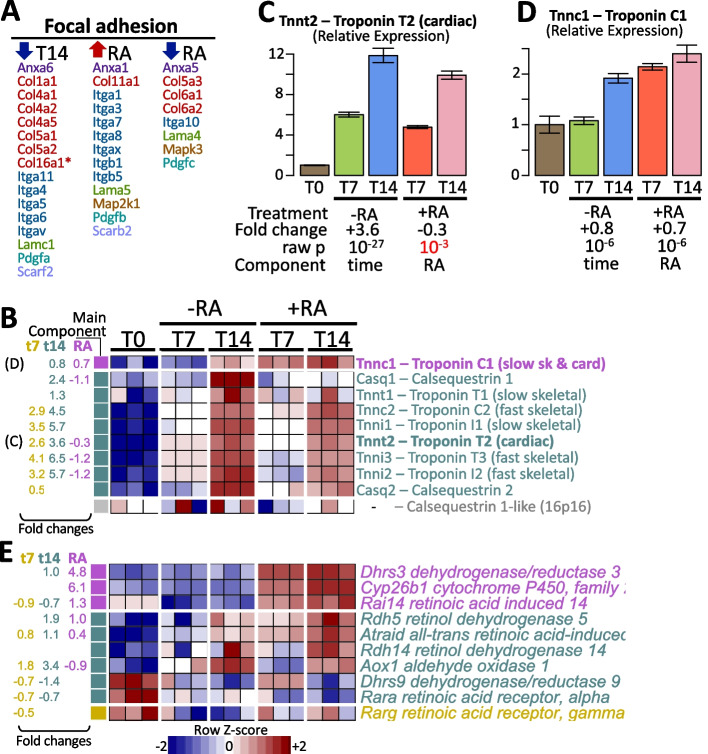
Analysis of specific sets of genes. **A** The genes associated with focal adhesion from Fig. 4. **B** Selected markers from Branco et al. 2015 showing that the influence of the RA component is minor. **C** Close view of cardiac troponin where the time component is the most influent factor. **D** Close view of slow cardiac and skeletal troponin where both time and RA components are factors of transcriptional changes. **E** Receptors and retinol metabolism KEGG rno00830 pathway (only those expressed and significant for any factor). +RA: with retinoic acid; -RA: without retinoic acid; T0: 24 h after seeding; T7: 7 days after seeding; T14: 14 days after seeding

To better understand the functional roles of the gene expression changes, we analyzed each of the six clusters independently using EnrichR [19], a functional enrichment tool. This tool estimates whether a list of genes contains more genes than expected by random chance for a set of genes, where a set of genes can be a pathway, a domain, genes related to disease, or any other functional concept. We used gene sets whose gene size was less than 200, contained more than 7 differential expressed genes, and whose FDR-adjusted *p*-value was < 10%. The functional concept that deactivates early at day 7 independently of RA (cluster *f*) is the *Epithelial to Mesenchymal Transition*, suggesting that cellular attachment is favored. Contrary, those that become active since day 7 (cluster *e*) involve *mTORC1 signaling* indicating preparation for cell growth and metabolism. Other early activations include the *Myoblasts* phenotype confirming a predisposition for muscle cells. Then, those concepts that gradually deactivate up to day 14 (cluster *d*) are related to functions decreasing cellular divisions (e.g., *spindle* and *DNA replication*). On the other hand, those that activate at day 14 independently of RA (cluster *c*) include *Muscle contraction* involving troponins and myosin genes, *Cardiac contraction* comprising mitochondrial complexes of cytochromes and Ca+ channels, and other mitochondrial concepts for energy generation. In agreement with our cellular characterization shown in Fig. 2, these transcriptional activating concepts support the RA-independent predisposition of H9c2 cells to set down, generate energy and perform muscle and cardiac functions. Then, the concepts that activate specifically by RA treatment were *Myogenesis* (Troponin C1, Integrins A7/B1/B5, Myosin Light Chain Kinase, among others), *Integrins / Cell Adhesion*, and *Cardiomyopathy* related genes (Integrins A1/A3/A8/A7, B1/B5, TGFB2, Troponin C1), and *elastic fibers formation* (Fibulin 1, Latent transforming growth factor beta binding protein 1/3, Lysyl Oxidase Like 2, and Integrins). On the contrary, concepts deactivating by RA treatment are related to *cell motility*, *membrane*, and *caveolae*. We noted that the *focal adhesion* concept was significant in several clusters (downregulated in 14d and RA and upregulated in RA) and composed mainly of annexins, collagens, integrins, laminins, and platelet-derived growth factors. Nevertheless, each cluster showed specific genes up and down-regulated (Fig. 5A).

### Key markers response to retinoic acid are different than previously thought

Previous studies have suggested that Troponin T, I, and calsequestrin are cardiac markers induced by using RA in the medium [1]. Nevertheless, our analysis shows that most of the genes of these gene families are indeed strongly induced by culture time rather than by RA (Fig. 5B). For example, Troponin T2 increased the expression 2.6 times at 7 days and 3.6 times at 14 days but also seemed slightly reduced under RA by -0.3 times (Fig. 5C). In these gene families, we only noted a specific modest increase due to RA in Troponin C1 (by +0.7 times, Fig. 5D). Also, a decrease was observed in Calsequestrin 1 under RA (Casq1, -1.1 folds) on top of the time increase (+2.4 folds, Fig. 5B).

One would expect genes involved directly in RA metabolism to show some changes during the stimuli. Thus, we focused on the RA-related genes (Fig. 5E, receptors and retinol metabolism KEGG rno00830 pathway, only those genes expressed and changing in any condition). We noted *Dhrs3*, *Cyp26b1* were highly upregulated, both directly involved in RA metabolism. We also observed an increase in the RA treatment *Rai14*, *Rdh5*, and *Atraid* (RA-induced 14, retinol dehydrogenase 5, and all-trans RA-induced, respectively). Only *Rai14* was strongly responsive to RA (+1.3 times in average at days 7 and 14) and indeed showed some decrease by time independently of RA (-0.9 times at 7 days and -0.7 times at 14 days). *Aox1*, also involved in the RA pathway, increased strongly over time (to +1.8 at 7 days and +3.4 times at 14 days) but decreased under RA (-0.9 times in average up to 14 days). RA receptors, however, (alpha and gamma) decreased their expression significantly over time (*Rara* by -0.7 times and *Rarg* by -0.5 times), and non-significant changes were obtained under RA. Beta RA receptor was not observed expressed in our experiments. Many other genes of the RA pathway were expressed but not differentially in any condition (*Adh5, Rarres2, Pnpla4, Ugt1a6, Dgat1, Retsat, Bco1, Rxra, Aox2, Rdh10, Rxrb, Rai1, Rdh13, Dhrs4, Rdh11*). Contrary to Branco et al. [1], we did not find retinol-binding protein 2 (*Rbp2*) expression in any sample.

**Fig. 6:**
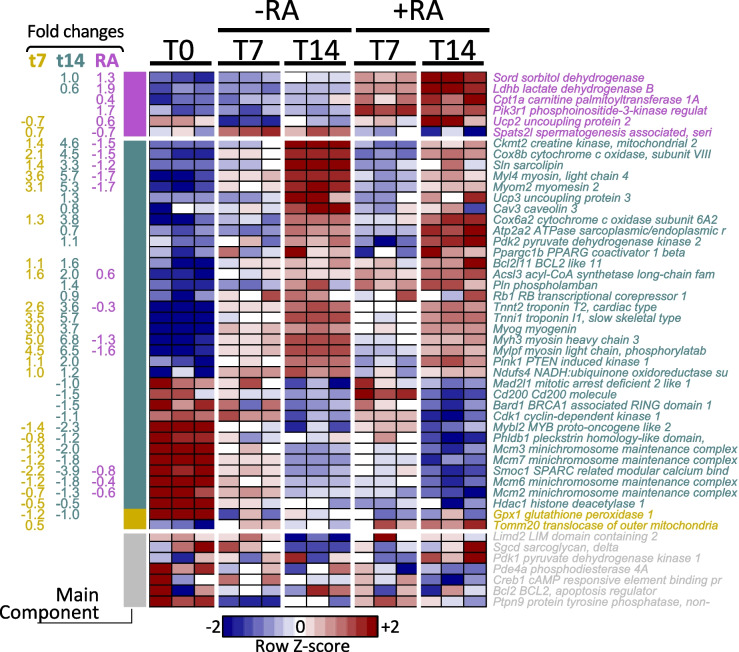
Our results for selected genes from previous reports. Only significant factors are expressed as fold changes at the left. Only 6 genes (top) appear to respond to RA stronger than time in the previously reported genes. In summary, most genes associated to RA in previous reports are associated to time rather than RA. +RA: with retinoic acid; -RA: without retinoic acid; T0: 24 h after seeding; T7: 7 days after seeding; T14: 14 days after seeding

Previous studies suggested essential and selected RA-induced genes [1, 9] covering 70 unique genes (Table 1 and Figures 2 & 3 in [1], and Table 2 and Figure 2 in [9]). From there, we found 49 genes estimated to be expressed in our RNA-Seq experiments, of which 42 have been differentially expressed at a raw *p* < 10^–3^ (Fig. 6). From the 49 genes, only 6 responded more strongly to RA (*Sord*,* Ldhb*,* Cpt1a*,* Pik3r1*,* Ucp2*, and *Spats2l*), and 12 more genes have a minor additive RA response (*Ckmt2*,* Cox8b*,* Sln*,* Myl4*,* Myom2*,* Acsl3*,* Tnnt2*,* Myh3*,* Mylpf*,* Smoc1*,* Mcm6*, and *Mcm2*) often contrary in sign to the time response. Thus, our results suggest that most of the previously reported responses are due to culture conditions over time rather than to the RA.

## Discussion

Previous studies suggested that H9c2 cells, highly used to analyze cardiac responses in vitro, responded to RA stimuli by changing the expression of more than 3000 genes [1, 9]. Nevertheless, previous studies have shown that around 350 DNA loci respond to RA in a particular cell type and aggregate up to 700 genes across different cell types [8]. Here, by performing an experimental design specific for RA response over time, we showed that 622 genes responded to RA, of which 440 genes showed a stronger response than culture time alone and 305 were specific for the RA stimuli. These results have implications for the interpretation of the role of RA in assays regarding H9c2 cells, specifically for cardiology and cell differentiation.

One key feature of successfully differentiating myoblast cells is the modification of cell morphology, where differentiated cells are characterized as larger multinucleated cells in a closer relation with cardiac like-myotubes. Related to this, RA has been argued as a crucial effector in achieving this phenotype [1, 5, 20]. However, the RA-based differentiation protocol reveals inefficient as it has been consistently reported to only differentiate a portion of the total cell population. Related to this, the most striking observation we made during our study was that time is the single most important factor in generating multinucleated myotube-like cells rather than RA. In fact, looking only into cell morphology, RA only contributed to promoting an increase in cell fusion and, therefore, the complexity of already multinucleated cells after 14 days, thus modifying the proportion between 2-nuclei cells and 3 nuclei cells, while mononucleated cells do not seem to change at 14 days between cultures with or without RA. On the other hand, time allow the differentiation of more than 50% of cell regardless of the presence of RA. This does not mean that RA is not fitted to be used to differentiate cells, as it contributes at the molecular level to a more cardiac-like myotube phenotype. The reflection to be made is regarding if the protocol is efficient enough and the importance RA has over it. It has been noted that 3D cultures may facilitate the formation of multinucleated H9c2 cells [21]. Thus, our observation can contribute to the study of multinuclear cell formation [22], in which RA seems to be an important accelerating factor. Another observation that corroborates the importance of time in cell differentiation is the increase of cell area that, in this case, can be considered a surrogate of increased cell size by fusion rather than hypertrophy, as differentiated cells acquired an elongated shape. To this matter, we found that RA is not interfering and contributing to the increase of cell area, but over time cell area increased. This can be correlated with the observation made by Kankeu et al. [9], where differentiated cells using the same protocol showed to spread to a higher length and a shorter diameter, leading to an increased length-to-diameter ratio and, therefore, area, as a marker of the differentiated cell population.

Partial multinucleated cells during H9c2 + RA stimulation have been observed by Branco et al. Here, we showed that multinucleated phenotype is present even without the RA stimuli but also that RA stimuli almost double the presence of multinucleated cells. We also observed that the mononucleated cells do not seem to change at 14 days between cultures with or without RA. It has been noted that 3D cultures may facilitate the formation of multinucleated H9c2 cells [21]. Thus, our observation can contribute to the study of multinuclear cell formation [22], in which RA seems to be an important accelerating factor.

Gene expression of cardiac proteins, such as SERCA, RyR, and L-type Ca2+ channels, increase their levels by RA treatment, which has been proved previously [1, 5], indicating a preferred differentiation to a cardiac-like phenotype. One important difference is that Branco et al. studied the characterization of cardiomyoblast H9c2 cell line after cells reached 80% confluence then exposed to RA for 7 days. In our study, we compare differentiated myotubes exposed to RA through the time since the first day of culture, exploring the implication of each variable to a cardiac phenotype, confluence, time, and RA. Other authors have explored the differentiation protocol, observing the myotubes formation at 5 days. Ménard et al. followed the differentiation protocol for 10 days finding a time-depending change in L-type Ca2+ channel expression. Szalai et al. found cardiac and skeletal L-type Ca2+ involved in cytosolic Ca2+ response without RA treatment. Despite the fact that after 5 days of the differentiation process, a myotube can be obtained, we found that time is a crucial factor for a sustained cardiac-like phenotype. It is optimal to study the myotubes for a longer time course to have a cell model more morphologically and molecularly approached to a cardiac cell [1, 5, 12]. However, regardless of the key role of these proteins in intracellular calcium handling, the response to RyR stimulation in RA-treated cells is not greater than in control cells. Previous reports showed an important decreased response to vasopressin due to the depletion of intracellular Ca2+ stores evoked by caffeine [12, 23] after time differentiation. RA treatment showed a similar trend, decreasing the Ca2+ released by vasopressin after caffeine stimulation; however, by T14, both groups manage a similar caffeine/VP Ca2+ release ratio, suggesting that elements related to cardiac-like differentiation phenotype are more likely linked to time than RA treatment. Regarding energetic metabolism, we observed both transcriptional and functional upregulation of mitochondrial oxidative metabolism during the differentiation process, indicating that cells have a more oxidative metabolic shift. However, this occurs independently of retinoic acid in the differentiation media, although retinoic acid exerts a stronger stimulating effect. In this regard, it is interesting to note that levels of the rate-controlling enzyme of β-oxidation, CPT1, are highly upregulated during the differentiation processes indicating that differentiated cells shift their mitochondrial metabolism to fatty acid oxidation and this effect, although also observed in the absence of retinoic acid, is enhanced by agonists and substrates of the retinoid X receptors (RXR) that jointly act with PPAR to activate oxidative metabolism [24].

An interesting observation is that the RA response is a mixture of time and the RA stimuli from those genes that responded to RA; 440 (70%) genes already changed at 7 days and sustained to day 14. It would be exciting to understand the dynamic cascade of gene regulation during the first 7 days either by shortening the harvesting for RNA-Seq and/or by single-cell RNA-Sequencing. From those 182 genes that responded to RA but whose culture time factor was stronger, we noted a provoking effect in ~ 70% of the genes in which the coefficient sign for RA was opposite to the culture time coefficient. For these genes shown in Fig. 4 (marked with *), gene expression on day 7 without RA was similar to the gene expression on day 14 under RA treatment. This can be interpreted as a “delay effect” in the gene expression response. However, it is unknown whether it is a transitory state that could be established in larger culture times favoring the delay effect or a stable result of two opposite transcriptional forces. In any case, experiments sampling more often at early culture times and larger than 14 days would reveal more details about the gene expression dynamics.

A recent multi-species meta-analysis study of the transcriptional response was published while this manuscript was in preparation [25]. The authors used data derived from cell lines from chicken (LCM hepatocellular carcinoma), human (SH-SY5Y neuroblastoma), mouse (embryonic stem cells and leukemia model BCR-ABL1), and frog (*Xenopus laevis* pancreatic explants). They showed that 91 genes were differentially expressed under RA treatment and that all of them were upregulated. Contrary, we observed a similar number of genes up- and down-regulated under RA (Fig. 4) on days 7 and 14. We noted that the culture times of most of the experiments performed in the Falker-Gieske et al. meta-analysis were short (from 2 h to 2 days) compared to our experiments (sampled at 7 and 14 days). Thus, it is possible that rapid transitory responses could be enriched in transcriptional activations and that our results reflect the cascade of a secondary transcriptional and more stable state.

## Conclusions

Our study demonstrated that RA is not the single neither the most influential factor for H9C2 cell differentiation into cardiac myoblast phenotype. Otherwise, time-dependent differentiation is found to generate a sustained change toward cardiac phenotype. At the molecular level, RA contributes specifically to a fraction of transcriptome modifications, including changes in intracellular calcium signaling towards cardiac-like myoblast, although functional responses of cells are not sustained over culture time. Finally, these findings suggest that H9C2 differentiation protocols into cardiac-like myoblast required a deeper understanding and possible reformulation, where RA is only one player to take into account.

## Supplementary Information

**Additional file 1: Supplementary Figure 1.** Original western blots gel images. Top gel shows the two digital cuts shown in main manuscript for the labeled calcium channels. Bottom gel shows the corresponding digital cut for the beta-actin protein.

## Data Availability

The raw counts after mapping are available in the GEO database at NCBI by the id GSE221596 (https://www.ncbi.nlm.nih.gov/geo/query/acc.cgi?acc=GSE221596).
