# Supplementary material for: The retinoic acid response is a minor component of the cardiac phenotype in H9c2 myoblast differentiation
Source: BMC Genomics. 2023 Aug 2;24:431. doi: 10.1186/s12864-023-09512-0 (PMC10394869; doi:10.1186/s12864-023-09512-0)
Supplement: Supplementary file 1 — Additional file 1: Supplementary Figure 1. Original western blots gel images. Top gel shows the two digital cuts shown in main manuscript for the labeled calcium channels. Bottom gel shows the corresponding digital cut for the beta-actin protein. [file 12864_2023_9512_MOESM1_ESM.docx]

Supplementary Information

**The retinoic acid response is a minor component of the cardiac phenotype in H9c2 myoblast differentiation**

**Carlos Campero-Basaldua, Jessica Herrera-Gamboa, Judith Bernal-Ramírez, Silvia Lopez-Moran, Luis-Alberto Luévano-Martínez, Hugo Alves-Figueiredo, Guillermo Guerrero, Gerardo García-Rivas, Víctor Treviño**

**Supplementary Figure 1. *Original western blots gel images.*** Top gel shows the two digital cuts shown in main manuscript for the labeled calcium channels. Bottom gel shows the corresponding digital cut for the beta-actin protein.
